# Transcriptional Regulation of Channelopathies in Genetic and Acquired Epilepsies

**DOI:** 10.3389/fncel.2019.00587

**Published:** 2020-01-14

**Authors:** Karen M. J. van Loo, Albert J. Becker

**Affiliations:** Department of Neuropathology, Section for Translational Epilepsy Research, University of Bonn Medical Center, Bonn, Germany

**Keywords:** genetic and acquired epilepsies, ion channels, channelopathies, transcriptional regulation, neurodevelopmental disorders

## Abstract

Epilepsy is a common neurological disorder characterized by recurrent uncontrolled seizures and has an idiopathic “*genetic*” etiology or a symptomatic “*acquired*” component. Genetic studies have revealed that many epilepsy susceptibility genes encode ion channels, including voltage-gated sodium, potassium and calcium channels. The high prevalence of ion channels in epilepsy pathogenesis led to the causative concept of “ion channelopathies,” which can be elicited by specific mutations in the coding or promoter regions of genes in *genetic* epilepsies. Intriguingly, expression changes of the same ion channel genes by augmentation of specific transcription factors (TFs) early after an insult can underlie *acquired* epilepsies. In this study, we review how the transcriptional regulation of ion channels in both *genetic* and *acquired* epilepsies can be controlled, and compare these epilepsy “ion channelopathies” with other neurodevelopmental disorders.

## Introduction

Epilepsy is a severe chronic brain disorder characterized by recurrent seizure activity due to aberrant neuronal network activity (Fisher et al., 2014; Fisher, 2015). Despite many years of research, the underlying mechanisms that orchestrate seizure activity are still not fully understood. This is also reflected in the fact that treatment strategies for epilepsy with antiepileptic drugs (AEDs) are insufficient in about one-third of epilepsy patients (Kwan and Sander, 2004). This relatively high level of pharmacoresistance, together with the often severe side effects of AEDs, asks for a better understanding of its etiology and pathogenesis (Löscher et al., 2013).

Nowadays, it is generally accepted that both genetic as well as environmental factors, such as head trauma, brain tumors, brain infection, stroke, autoimmune diseases, status epilepticus (SE) and hippocampal sclerosis (Engel, 1996; Bien and Elger, 2007; Bien et al., 2007; Liu et al., 2016; Pitkänen et al., 2016; Vezzani et al., 2016) can play a role in the etiopathogenesis of epilepsy. Epilepsies with such a causal injury to the central nervous system (CNS) are called *acquired* or *symptomatic* epilepsies, whereas those lacking a clear predisposing cause, are called *idiopathic* or *genetic* epilepsies (Shorvon, 2011).

In the last decades, enormous progress has been made in the discovery of epilepsy genes, resulting in a current list of approximately 1,000 epilepsy-associated genes (reviewed by Wang et al., 2017). Since many of the genes annotated on this list are ion channels, the theory was born that epilepsy is a disease of “ion channelopathies” (Wallace et al., 1998; Reid et al., 2009). Ion channels are pore-forming membrane proteins involved in maintaining ion homeostasis and the generation and propagation of neuronal action potentials. A disturbance in the neuronal ionic flow might result in hyperexcitability, which can form the basis for seizure activity (Raimondo et al., 2015). In general, ion channels can be divided into two main groups, depending on their mode of activation (Brenowitz et al., 2017). Voltage-gated ion channels are activated by changes in membrane potential and ligand-gated ion channels are opened in response to specific ligands binding to the extracellular domain of the ion channel (Alexander et al., 2015a,b).

In this study, we focus on the transcriptional mechanisms involved in channelopathy-induced epilepsy. We review how the expression of ion channel genes can be affected and compare these mechanisms between *genetic* and *acquired* epilepsies. In addition, we also summarize how these transcriptional mechanisms can play a role in the etiopathogenesis of other neurodevelopmental disorders.

## Voltage-Gated and Ligand-Gated Ion Channels in Genetic Epilepsies

For decades, scientists try to unravel the molecular background of epilepsies. In 1995, the first epilepsy-associated ion channel was identified; a mutation in a strongly conserved amino acid residue in the acetylcholine receptor alpha 4 subunit (CHRNA4) correlated with autosomal dominant nocturnal frontal lobe epilepsy (ADNFLE; Steinlein et al., 1995). After this first discovery, many other ion channels were reported to be linked to epilepsy, including genes belonging to the voltage-gated sodium, potassium, calcium and hyperpolarization-activated cyclic nucleotide-gated (HCN) channels. Besides the voltage-gated ion channels, also several ligand-gated ion channel genes were identified as epilepsy-associated genes, including ionotropic glutamate receptors, GABA_A_ receptors and nicotinic acetylcholine receptors (Table 1; reviewed by Lerche et al., 2013; Wang et al., 2017; Wei et al., 2017; Oyrer et al., 2018).

**Table 1:** Transcriptional channelopathies implicated in epilepsies.

Gene*	Protein	Transcriptional mechanisms	Impact	References
**Sodium channels**				
*Scn1a*	Na_v_1.1	Genetic variants in the promoter region	LOF	Nakayama et al. (2010) and Gao et al. (2017)
		Differential TF-binding	LOF	Dong et al. (2014)
		Epigenetic control mechanisms	LOF	Schuster et al. (2019)
		Alternative splicing	LOF	Lossin (2009) and Schlachter et al. (2009)
*Scn1b*	Na_v_β1			
*Scn2a*	Na_v_1.2	Differential TF-binding	LOF	Xiang et al. (2018)
*Scn3a*	Na_v_1.3	Epigenetic control mechanisms	GOF	Li et al. (2015) and Tan et al. (2017)
*Scn8a*	Na_v_1.6	Epigenetic control mechanisms	GOF	Liu et al. (2017)
*Scn9a*	Na_v_1.7	Differential TF-binding	GOF	Diss et al. (2008)
**Potassium channels**				
*Kcna1*	K_v_1.1	Regulation by miRNAs	LOF	Sosanya et al. (2013)
*Kcna2*	K_v_1.2			
*Kcnb1*	K_v_2.1			
*Kcnc1*	K_v_3.1			
*Kcnd2*	K_v_4.2	Differential TF-binding	LOF	Gross et al. (2016) and Tiwari et al. (2019)
		Regulation by miRNAs	LOF	Yao et al. (2016)
*Kcnd3*	K_v_4.3			
*Kcnh2*	K_v_11.1			
*Kcnh3*	K_v_12.2			
*Kcnh5*	K_v_10.2			
*Kcnj10*	Kir4.1	Epigenetic control mechanisms	LOF, GOF	Nwaobi et al. (2014) and Zhang et al. (2018)
*Kcnk10*	K2P10.1	Regulation by miRNAs	GOF	Haenisch et al. (2016)
*Kcnq2*	K_v_7.2	Differential TF-binding	LOF	Mucha et al. (2010)
		Alternative splicing	LOF	de Haan et al. (2006)
*Kcnq3*	K_v_7.3	Differential TF-binding	LOF	Mucha et al. (2010)
*Kcnv2*	K_v_8.2			
*Kcnma1*	K_ca_1.1			
*Kcnt1*	K_ca_4.1			
*Kctd7*	Kctd-7			
**Calcium channels**				
*Cacna1a*	Ca_v_2.1			
*Cacna1g*	Ca_v_3.1			
*Cacna1h*	Ca_v_3.2	Alternative splicing	GOF	Powell et al. (2009)
		Differential TF-binding	GOF	van Loo et al. (2012, 2015)
*Cacna2d2*	Ca_v_α2Δ-2			
*Cacna2d4*	Ca_v_α2Δ-4	Differential TF-binding	GOF	van Loo et al. (2019)
*Cacnb4*	Ca_v_β4			
**Chloride channels**				
*Clcn2*	Clc-2	Alternative splicing	LOF	Bertelli et al. (2007)
*Clcn4*	Clc-4	Alternative splicing	LOF	Palmer et al. (2018)
**GABA_A_ receptors**				
*Gabra1*	GABA_A_α1	Differential TF-binding	LOF	Hu et al. (2008); Lund et al. (2008)
		Epigenetic control mechanisms	LOF	Bohnsack et al. (2017)
*Gabra4*	GABA_A_α4	Differential TF-binding	GOF	Roberts et al. (2005)
*Gabra6*	GABA_A_α6			
*Gabrb1*	GABA_A_β1	Differential TF-binding	LOF	Li et al. (2018)
*Gabrb2*	GABA_A_β2			
*Gabrb3*	GABA_A_β3	Genetic variants in the promoter region	LOF	Urak et al. (2006)
		Differential TF-binding	LOF	Tanaka et al. (2012a)
		Epigenetic control mechanisms	LOF	Tanaka et al. (2012a); Tanaka et al. (2012b)
*Gabrd*	GABA_A_δ			
*Gabrg2*	GABA_A_γ2	Alternative splicing	LOF	Kananura et al. (2002)
**Ionotropic glutamate receptors**				
*Gria2*	GluA2	Epigenetic control mechanisms	LOF	Machnes et al. (2013) and Kiese et al. (2017)
*Grin1*	GluN1			
*Grin2a*	GluN2A	Epigenetic control mechanisms	GOF	Kiese et al. (2017)
		Regulation by miRNAs	GOF	Alsharafi et al. (2016)
*Grin2b*	GuN2B	Epigenetic control mechanisms Alternative splicing	LOF undetermined	Parrish et al. (2013) Smigiel et al. (2016)
*Grin2d*	GluN2D			
**Nicotinic acetylcholine receptors**				
*Chrna2*	nAChRα2			
*Chrna4*	nAChRα4			
*Chrna7*	nAChRα7			
*Chrnb2*	nAChRβ2			
**Hyperpolarization-activated cyclic nucleotide-gated channels**				
*Hcn1*	Hcn1	Epigenetic control mechanisms	LOF	McClelland et al. (2011)
*Hcn2*	Hcn2			
*Hcn4*	Hcn4			

*Epilepsy genes based on Online Mendelian Inheritance in Man (OMIM) database, Wang et al. (2017), Wei et al. (2017) and Oyrer et al. (2018). LOF, Loss-of-function; GOF, Gain-of-function; TF, Transcription Factor; miRNA, microRNA.

Currently, it is thought that *genetic* epilepsy can be the result of: (i) rare variants with high penetrance (also known as monogenic or “common-disease-rare-variant model”) or of (ii) common variants with low penetrance (also known as polygenic or “common-disease-common-variants model”; Reich and Lander, 2001; Gibson, 2012; Saint Pierre and Génin, 2014). Such rare variants (or mutations) can nowadays be identified by deep sequencing approaches (e.g., exome sequencing or whole-genome sequencing; Dunn et al., 2018), whereas for the identification of common variants (also known as single nucleotide polymorphisms, SNPs), genome-wide association studies are indispensable in large cohorts of patients and controls (International League Against Epilepsy Consortium on Complex Epilepsies, 2018). However, common variants are often difficult to link unequivocally to disease, since these variants contribute only minimally and might also require an additional environmental factor for a pathological outcome.

In epilepsy, both rare as well as common variants have been identified in ion channel genes. Mutations in the sodium channel *SCN1A*, probably the most studied and best-documented epilepsy gene, can cause a spectrum of epilepsy syndromes including Dravet syndrome and genetic (generalized) epilepsy with febrile Seizures Plus (GEFS+; Brunklaus and Zuberi, 2014), whereas a common variant within an intron of the same gene (rs7587026), was found to associate with mesial temporal lobe epilepsy (mTLE; Kasperaviciute et al., 2013).

Mostly, *genetic* channelopathies are the result of variants within the coding region of the gene. Both missense mutations (mutations causing an amino acid change), as well as nonsense mutations (mutations causing a premature stop codon), can underlie epilepsy pathogenesis by inducing a loss-of-function (LOF) or a gain-of-function (GOF) channelopathy. In addition, also deletions and duplications of (part of) the gene can strongly affect normal channel function (Borlot et al., 2017; Monlong et al., 2018). Since the focus of this review is on the transcriptional regulation of ion channels, listing all genetic variants within the coding regions of ion channel genes is beyond the scope of this article (for reviews, see Deng et al., 2014; Wei et al., 2017; Zhang et al., 2019).

## Transcriptional Regulation of Genetic Ion Channelopathies

*Genetic* epilepsies can also be the result of a genetic variant positioned in the promoter region, a splice site, or a regulatory region of the gene. How can variants outside the coding region induce a channelopathy? For *SCN1A* for example, a microdeletion in the 5’-promoter region was found in patients with Dravet syndrome (Nakayama et al., 2010), and another heterozygous mutation in the promoter region (h1u-1962 T >G) was identified in a patient with partial epilepsy and febrile seizures (Gao et al., 2017). Functional analysis of this *SCN1A* h1u-1962 T >G variant revealed a reduction of *SCN1A* promoter activity by 42.1% compared to the wild-type variant (Gao et al., 2017), explaining the relatively mild phenotypical impairment caused by this non-coding variant when compared with effects caused by *SCN1A* coding variants that can result in *null* expression.

A genetic variant can also be located at a splice site, resulting in alternative splicing of the ion channel gene. This process allows a single gene to produce alternative ion channels with different functional characteristics. In particular, for *SCN1A* many alternative splicing mutations have been identified in epilepsy pathogenesis (Lossin, 2009; Thompson et al., 2011; Carvill et al., 2018; Table 1).

If a genetic variant is located within the binding site of an activating or repressing transcription factor (TF) or a repressor, it may result in altered regulation of the transcriptional machinery. For example, four different haplotypes, consisting of 13 SNPs located in the 5’ region of the *GABRB3* gene were found to segregate with childhood absence epilepsy (CAE; Urak et al., 2006). The *GABRB3* gene encodes the β3 subunit of the GABA_A_ receptor which mediates phasic (synaptic) and tonic (perisynaptic) inhibition (Farrant and Nusser, 2005; Hirose, 2014). Functional analysis of these haplotypes revealed a reduced transcriptional activity of the *GABRB3*-haplotype-2 promoter (overrepresented in CAE) compared to the *GABRB3*-haplotype-1 promoter (overrepresented in controls). The difference in expression could be explained by reduced binding of the TF N-Oct3 to the *GABRB3*-haplotype-2 promoter, resulting in decreased expression of the *GABRB3* gene (Urak et al., 2006). The reduced β3 levels observed in CAE patients might cause a loss of inhibitory properties of the receptor, eventually causing seizure activity.

## Ion Channels in Acquired Epilepsies

*Acquired* epilepsies are epilepsies, which are on the consequence of an environmental factor. These epilepsies can be: (i) completely dependent on environmental factors; or (ii) can be caused by an interaction of environmental factor(s) with a predisposition genome. In the latter case, the presence of common susceptibility variants (e.g., SNPs or CNVs) can lower the threshold of the environmental factor for inducing an epileptic outcome. Most of our current knowledge of *acquired* epilepsy pathogenesis comes from the use of animal models, in which insults causing TLE can be mimicked in rodents using approaches like traumatic brain injury, kindling, or by applying one of the chemo-convulsants pilocarpine or kainic acid to induce SE (reviewed by Jefferys et al., 2016; Lévesque et al., 2016; Becker, 2018; Nirwan et al., 2018). Numerous studies using animal models for TLE have provided valuable information on epilepsy pathogenesis, resulting in a list of several channels involved in *acquired* epilepsies, including but not limited to HCN channels (Chen et al., 2001; Shah et al., 2004; Marcelin et al., 2009; Jung et al., 2011; Arnold et al., 2019), the A-type potassium channel K_v_4.2 (Bernard et al., 2004; Monaghan et al., 2008), K_ir_2 channels (Young et al., 2009), small-conductance (SK) calcium-activated potassium channels (Oliveira et al., 2010), big potassium channels (BK-channels; Pacheco Otalora et al., 2008; Shruti et al., 2008), persistent sodium channels (Agrawal et al., 2003; Chen et al., 2011), the T-type calcium channel Ca_V_3.2 (Su et al., 2002; Becker et al., 2008) and the calcium channel subunit α2δ4 (van Loo et al., 2019).

## Transcriptional Regulation of Acquired Ion Channelopathies

Currently, one of the main questions in epilepsy research is how the expression of ion channel genes in *acquired* epilepsies can be regulated. The transcriptional regulation of ion channels in *acquired* epilepsy can occur for example *via* differential expression of transcriptional activators or repressors. After a brain insult, a transient increase of activity-regulated TFs is evident (e.g., *Egr-4*, *Fos*, *Jun* and *Arc*), which can as a consequence dysregulate the transcriptional machinery of many genes, including ion channel genes (Herdegen et al., 1993; Beer et al., 1998; Herdegen and Leah, 1998; Honkaniemi and Sharp, 1999). To date, several transcriptional mechanisms have already been identified in the context of channelopathies and epilepsy pathogenesis (Table 1).

For Ca_V_3.2, we recently performed an in-depth promoter analysis, examining the molecular mechanisms involved in the transcriptional augmentation of this channel early after pilocarpine-induced SE (Becker et al., 2008). Here, we observed a highly-sophisticated mechanism of transcriptional regulation: the increase of Ca_V_3.2 expression was found to be mediated by metal-regulatory transcription factor 1 (MTF1) upon a rise in intracellular zinc ([Zn^2+^]_i_); denoted as the Zn^2+^-MTF1-Ca_V_3.2 cascade (van Loo et al., 2015). A rise in [Zn^2+^]_i_, often seen after a transient insult to the brain (Assaf and Chung, 1984; Zhao et al., 2014), can activate MTF1, which then binds to metal-responsive elements within the *Ca_V_3.2* promoter region. Consequently, this results in increased Ca_V_3.2 expression, a larger T-type current and increased burst-firing behavior (van Loo et al., 2015). In this way, the Zn^2+^-MTF1-Ca_V_3.2 cascade can enhance hippocampal network excitability, resulting in seizure activity. Besides the Zn^2+^-MTF1-Ca_V_3.2 cascade, also other TFs were found to control Ca_V_3.2 expression, including Egr1 and RE1-silencing transcription factor (REST; van Loo et al., 2012). Such a multifactorial regulation by several TFs, thought to be a general phenomenon of ion channel regulation, severely complicates pharmacological intervention.

## Epigenetic Control of Acquired Ion Channelopathies

The transcription of ion channels in *acquired* epilepsies can also be regulated at the epigenetic level: both DNA methylation at cytosine residues, as well as changes in histone modifications (e.g., acetylation or methylation) can strongly affect the transcriptional machinery (reviewed by Hauser et al., 2018). Methylation of DNA generally occurs at cytosines within the 5’-cytosine-guanine-3’ context (CpG). Gene promoters often contain large clusters of CpGs (referred to as CpG islands), which are mostly hypomethylated and are linked to transcriptional activation. An increase in DNA methylation may cause reduced transcriptional activity due to physical inhibition of TF binding to their cognate DNA binding motif, or by binding of repressor proteins known as methyl-CpG binding domain proteins (MBDs) to the methylated DNA. In the latter case, MBDs can recruit histone deacetylases (HDAC1 and HDAC2) to the methylated DNA, resulting in the silencing of the corresponding gene (Clouaire and Stancheva, 2008). To date, several epilepsy-channelopathies have been described to be caused by an epigenetic control mechanism (Table 1).

## Regulation of Acquired Ion Channelopathies by Micrornas

The transcriptional machinery of ion channels in *acquired* epilepsies can also be influenced by small non-coding RNAs, such as microRNAs (miRNAs). miRNAs are 22 nucleotides noncoding RNAs that can regulate gene expression by associating with the RNA-induced silencing complex (RISC). The RISC complex then uses the miRNA as a template for recognizing the complementary mRNA of the ion channel gene (Ranganathan and Sivasankar, 2014). The main function of miRNAs appears to be the regulation at the post-transcriptional level: either by hindering protein translation or by enhancing mRNA degradation. Nowadays, it is also debated that miRNAs can have a nuclear function by modulating gene expression directly at the transcriptional level (reviewed by Catalanotto et al., 2016). Numerous miRNAs have been identified in relation to epilepsy pathogenesis (reviewed by Reschke and Henshall, 2015; Henshall et al., 2016; Shao and Chen, 2017; Tiwari et al., 2018), and also several ion channels appear to be under control of miRNAs, including K_v_1.1, K_v_4.2, Kcnk10 and Grin2a (Sosanya et al., 2013; Alsharafi et al., 2016; Gross et al., 2016; Haenisch et al., 2016; Tiwari et al., 2019; Table 1).

## Transcriptional Regulation of Channelopathies in Neurodevelopmental Disorders

To date, it is generally accepted that ion channelopathies are not unique for epilepsy pathogenesis, but have gained considerable attention for the pathogenesis of several neurodevelopmental disorders, including pathology aspects of autism spectrum disorders (ASDs), schizophrenia, bipolar disorder, major depressive disorder and migraine (reviewed by Imbrici et al., 2013; Schmunk and Gargus, 2013; Albury et al., 2017). Seizures have been noted as a comorbidity feature of neurodevelopmental disorders (Hyde and Weinberger, 1997; Canitano, 2007; Mula et al., 2008; Liao et al., 2018; Salpekar and Mula, 2018; Strasser et al., 2018), which overall may point to the emergence of a functionally impaired neuronal network. For many neurodevelopmental disorders, several genetic variations (both rare mutations as well as common variants) in the coding regions of ion channel genes have been identified and reviewed elsewhere (Imbrici et al., 2013; Schmunk and Gargus, 2013; Daghsni et al., 2018; Weiss and Zamponi, 2019). Interestingly, also the mechanisms described above to be involved in the transcriptional regulation of ion channels in epilepsy pathogenesis, have been observed in the regulation of ion channels in other neurodevelopmental diseases. For example, transcriptional regulation by presence of genetic variants within the promoter region was observed for *Grin2a* and *Grin2b* and resulted in schizophrenia pathogenesis (Miyatake et al., 2002; Itokawa et al., 2003; Liu et al., 2015); alternative splicing was documented for *Gabrb2*, *Grin2b* and *Gabra3* and resulted in mental retardation and ASD (Zhao et al., 2009; Endele et al., 2010; O’Roak et al., 2012; Piton et al., 2013); differential expression of TFs was observed for *Scn10a*, *Kcnq1*, *Cacna1c*, and *Grin1* in schizophrenia and other psychiatric disorders (Rannals et al., 2016; Billingsley et al., 2018; Page et al., 2018); epigenetic control mechanisms were described for *Gabrb2*, *Gabrb3*, *Gria*, and *Chrna7* in ASD, schizophrenia and Rett syndrome (Samaco et al., 2005; Yasui et al., 2011; Kordi-Tamandani et al., 2013; Zong et al., 2017) and regulation by miRNAs was observed for *Cacna1c*, *Cacnb1*, *Grin2b* and NMDAR in schizophrenia and ASD (Kocerha et al., 2009; Guan et al., 2014; Cammaerts et al., 2015; Zhang et al., 2015; Kichukova et al., 2017).

Many of these “transcriptional channelopathies” apparently are rather specific, since they are mostly associated with only one individual neurodevelopmental disorder. However, a few examples exist, in which a comparable transcriptional regulatory mechanism has been observed for channelopathies in epilepsy and other neurodevelopmental disorders, hinting at an explanation for the comorbidity seen between the different disorders. One such example is the *GABRB3* gene, an important neurodevelopmental gene and besides epilepsy also associated with Angelman syndrome, Rett syndrome and ASD (Tanaka et al., 2012b). Differential expression of *GABRB3* in CAE can be caused by genetic variants within the promoter region (Urak et al., 2006). Interestingly, one of these variants is also associated with schizophrenia and heroin dependence (Chen et al., 2014b; Liu et al., 2019), whereas other genetic variants within the same regulatory region are correlated with ASD (Chen et al., 2014a).

Another example of comparable transcriptional control mechanisms in epilepsy and neurodevelopmental disorders was observed for the NMDAR gene *Grin2b*. The expression of *Grin2b* was significantly decreased in the kainic acid-induced SE model and correlated with increased DNA methylation levels at specific CpGs located within the *Grin2b* locus. Additionally, interfering with the DNA methylation levels prior to SE using a DNA-methyltransferase inhibitor, prevented the *Grin2b* DNA methylation increase after SE and resulted in augmented *Grin2b* mRNA and protein expression (Parrish et al., 2013).

Such a glutamatergic hypofunction caused by an epigenetic control mechanism in *Grin2b* in the epilepsy model can also contribute to the pathophysiology of other neurodevelopmental disorders (Coyle et al., 2002; Lau and Zukin, 2007). Recently, it was reported that in a mouse model for schizophrenia, *Grin2b* expression was also under control of epigenetic control mechanisms. Here, the reduction in *Grin2b* expression was caused by an increase in H3K27me3 and REST at the *Grin2b* promoter (Gulchina et al., 2017). We may thus assume that different neurodevelopmental disorders are associated with a channelopathy with similar underlying transcriptional mechanisms.

Although we see comparable transcriptional mechanisms, no large overlap exists between the individual regulatory players in epilepsy pathogenesis and other neurodevelopmental disorders. Of course, this can also be explained by the fact that most specific mechanisms simply have not been analyzed in all neurodevelopmental disorders, or not in an analogous manner, making a direct comparison at the moment impossible. Further studies will reveal whether the altered diseases-associated expression of more proteins is based on a (partly) general underlying transcriptional phenomenon, possibly explaining the comorbidity between epilepsy and other neurodevelopmental disorders.

## Future Perspectives

In this study, we reviewed the mechanisms involved in the transcriptional regulation of channelopathies in *genetic* and *acquired* epilepsies. Although a large amount of data exists, our understanding of transcriptional mechanisms governing ion channel expression is far from complete and requires further detailed investigation, not only for epilepsy pathogenesis, but also for other neurodevelopmental disorders. A better understanding of the underlying mechanisms might result in the development of novel drugs and may provide opportunities for better-individualized treatment strategies.

## Author Contributions

Both authors contributed to the writing and editing of the manuscript.

## Conflict of Interest

The authors declare that the research was conducted in the absence of any commercial or financial relationships that could be construed as a potential conflict of interest. The handling Editor declared a past co-authorship with one of the authors AB.
